# Development of a Type 2 Diabetes Prediction Model Using Specific Health Checkup Data and Extraction of Predictive Factors

**DOI:** 10.3390/bioengineering13020194

**Published:** 2026-02-09

**Authors:** Kenichiro Shimai, Kazuki Ohashi, Teppei Suzuki, Ryota Konno, Ryuichiro Ueda, Masami Mukai, Katsuhiko Ogasawara

**Affiliations:** 1 Faculty of Health Care Sciences, Department of Clinical Engineering, Jikei University of Health Care Sciences, Yodogawa-ku, Osaka 532-0003, Japan; roulant.g@gmail.com; 2 Faculty of Health Sciences, Hokkaido University, N12-W5, Kita-ku, Sapporo 060-0812, Japan; k_ohashi@pop.med.hokudai.ac.jp (K.O.); suzuki.teppei@i.hokkyodai.ac.jp (T.S.);; 3 Faculty of Education, Hokkaido University of Education, Iwamizawa Campus, Midorigaoka, Iwamizawa 068-0835, Japan; 4 Graduate School of Health Sciences, Hokkaido University, N12-W5, Kita-ku, Sapporo 060-0812, Japan; 5 Division of Medical Informatics, National Cancer Center Hospital, 5-1-1, Tsukiji, Chuo-ku, Tokyo 104-0045, Japan; mmukai@ncc.go.jp; 6 Faculty of Engineering, Muroran Institute of Technology, Mizumoto-cho, Muroran 050-8585, Japan

**Keywords:** health-checkups, type 2 diabetes mellitus, claims data, predictive model, logistic regression analysis

## Abstract

Background: Specific health checkups in Japan aim to prevent and detect non-communicable diseases (NCDs). Lifestyle information and non-invasive measurements obtained during these checkups are valuable for population health monitoring. This study aimed to develop a predictive model for type 2 diabetes mellitus (T2DM) using only non-invasive measurements and to identify key predictors. Methods: A retrospective observational study was conducted using linked health checkup records and medical claims from a city in Japan. Logistic regression was performed to predict a T2DM diagnosis. Results: A total of 409 of the 1363 participants were diagnosed with T2DM, including 285 of the 950 participants aged 40–74 years and 124 of the 413 participants aged ≥75 years. The model achieved an area under the receiver operating characteristic curve of 0.680 for those aged 40–74 years and 0.665 for those aged ≥75 years, indicating moderate discrimination. Key predictors included male sex, use of antihypertensive drugs, walking speed, and eating habits within 2 h before bedtime. In particular, male sex, having a slower walking speed, and not eating within 2 h before bedtime were positively associated with T2DM diagnosis. Conversely, the absence of antihypertensive or lipid-lowering medications was negatively associated with T2DM diagnosis. Conclusion: A model based solely on non-invasive measurements moderately identified individuals at risk for T2DM in this community-based Japanese population. Routinely collected health checkup data may support early identification and targeted preventive strategies.

## 1. Introduction

Diabetes mellitus, a representative non-communicable disease (NCD), has been increasing worldwide and remains a major public health concern in Japan [1]. The global prevalence of diabetes among individuals aged 20–79 years was estimated at 11.1% (589 million persons) in 2024, with one in nine adults affected. Prevalence is projected to rise to 13.0% (853 million persons) by 2050 [1]. The disease accounted for over 3.4 million deaths in 2024 and consumed more than one trillion US dollars in direct health expenditures [1]. According to a survey by the Japan Ministry of Health, Labour and Welfare, 16.8% of men and 8.9% of women are “persons strongly suspected of having diabetes”, with prevalence beginning to increase around age 40 and continuing to rise with advancing age [2]. The WHO Global Action Plan for the Prevention and Control of NCDs lists diabetes as one of its three priority target diseases [3]. Preventing and detecting diabetes at an early stage is therefore a critical public health priority both globally and in Japan.

Diabetes leads to serious complications, including cardiovascular disease and nephropathy [4], resulting in higher medical costs [5] and reduced quality-of-life [6]. To prevent such complications and improve community health, the early detection of type 2 diabetes mellitus (T2DM) is essential, as well as the identification of risk factors that can be linked to improved lifestyle habits. In Japan, regular health checkups, such as the health checkup program [7], are conducted to prevent NCDs and enable early detection. Regression analysis is frequently used to predict the risk of diseases including diabetes. In a large-scale multi-institutional joint study on Japanese workers, Nanri et al. used logistic regression analysis to formulate a straightforward risk score for predicting the three-year incidence rates of T2DM and developed a model that had a reasonably good predictive ability [8]. Ashizawa et al. highlighted the usefulness of lifestyle habit questions included in specific health checkups as predictors of metabolic syndrome in the subsequent year [9]. Furthermore, at the national level, robust machine learning models such as XGBoost and CatBoost have been developed to predict T2DM in specific regional populations. Although these models have demonstrated very high predictive performance, they required as many as 80 features, including multiple blood test parameters, which may limit their feasibility in broader community settings [10]. Research into predictive models using specific health checkups has progressed to date, but there has not been much research focusing on specific regional populations. In addition, developing non-invasive predictive models that do not require blood tests can contribute to improving T2DM prediction in targeted regional populations while relying on a limited set of features.

The data acquired during specific health checkups were questionnaire response results, measurement results including physical measurements, and the results from blood tests. Blood tests provide data used as diagnostic standards for diabetes, including hemoglobin A1C (HbA1c) values and fasting blood glucose levels. Questionnaire responses provide non-invasive data. In large-scale screening, non-invasive data are advantageous owing to their cost-effectiveness and ease of acquisition compared with invasive methods [11]. To ensure that health checkups generate cost-effective outcomes, it is crucial to establish a targeting strategy that determines which examinees should receive additional interventions or follow-up [12]. Therefore, the aim of this study was to develop a predictive model for T2DM using non-invasive parameters obtained from health checkups and to identify related predictive factors in a regional Japanese population.

## 2. Materials and Methods

### 2.1. Study Population and Database

This study used specific health checkups and health insurance claim data obtained from a single municipality (population: <80,000 persons) in Hokkaido. To conduct the analysis using health insurance claims data from different age groups, two sets of data were used: the National Health Insurance dataset (NHID) covering individuals aged 40–74 years and the Medical Care System dataset covering older adults aged ≥75 years (MCSD). To obtain the National Health Insurance data, ID linkage was implemented to connect the specific health checkup data of 6917 patients in fiscal years (FY) 2017 and 2018 and health insurance claims data for FY 2019. This process resulted in the identification of 285 individuals diagnosed with T2DM. Similarly, to obtain the Medical Care System for the Elderly Aged 75 and Over data, ID linkage was implemented to connect the specific health checkup data of 1854 patients in FYs 2017 and 2018 and the health insurance claims data for FY 2019. This process resulted in the identification of 124 individuals diagnosed with T2DM. In terms of the number of individuals who were not diagnosed with T2DM, 665 persons were identified from NHID, while 289 persons were identified from the MCSD (Figures S1 and S2). To determine the individuals eligible for analysis, the oldest data were used when specific health checkup data for multiple years were present; for health insurance claims, any duplicated data for the same individual was removed.

### 2.2. Statistical Analysis

Logistic regression analysis was performed using the presence or absence of a T2DM diagnosis as the objective variable, and explanatory variables such as sex, waist circumference, and body mass index (BMI). In our model, the “name of illness” for FY2019 (T2DM diagnosis, yes or no) was used as the objective variable, while the health checkup questionnaire items, sex, BMI, and waist circumference were used as the explanatory variables. Table 1 showed the questionnaire items and response options. Responses to the original questionnaire item “insulin injections and/or taking medicine for reducing blood sugar, yes or no” was used to confirm whether an individual is undergoing diabetes treatment at a medical institution and is actively taking medication [7]; this item was excluded as it solely determined a diabetes designation (yes or no). Furthermore, to identify the risk factors from the questionnaire items related to lifestyle habits, the following questions were asked: “history of stroke, yes or no”; “history of heart disease, yes or no”; “history of kidney failure, yes or no”; and “history of anemia, yes or no.” These questionnaire items were also excluded from the analysis of the present study. The present study aimed to extract the risk factors from the non-invasive data, especially prior to the medical intervention. Therefore, the results of blood tests were not used as a variable in this study, although they were utilized in previous studies. In the logistic regression analysis, the significance level was set at 0.05, and a 95% confidence interval was estimated for the partial regression coefficient and the odds ratio. An area under the receiver operating characteristic curve (AUROC) was used to evaluate the model. JMP Pro 17 (SAS Institute Inc., Cary, NC, USA) was utilized for consolidating the features to be analyzed; a chi-square test was used to analyze categorical variables, while a *t*-test was used to analyze continuous variables. IBM SPSS modeler 18.2 (IBM Corp., Armonk, NY, USA) was used for the logistic regression analysis.

## 3. Results

### 3.1. Participant Characteristics

The characteristics of the participants for NHID (persons diagnosed with diabetes: 285; persons not diagnosed with diabetes: 665) and those for MCSD (persons diagnosed with T2DM: 124; persons not diagnosed with T2DM: 289) were as shown in Table 2. In the aggregate results, comparisons between the two groups (persons diagnosed with or without T2DM) revealed distinctions within the NHID in the items “BMI”, “waist”, “antihypertensive drug”, and “lipid-lowering drug”, and within the MCSD in the items “sex” and “antihypertensive drug”.

### 3.2. Logistic Regression Analysis

**Figure 1:**
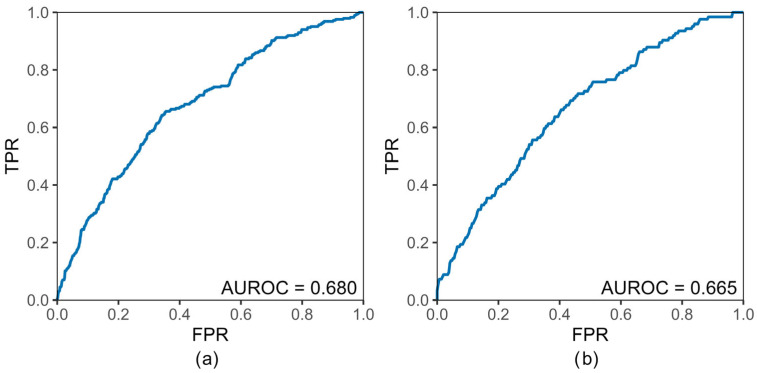
Results of the Receiver Operating Characteristic curve. Figure 1 showed the area under the receiver operating characteristic curve for (**a**) National Health Insurance dataset covering individuals aged 40–74 years and (**b**) Medical Care System dataset covering older adults aged ≥75 years (**right**).

The AUROC by logistic regression analysis was shown in Figure 1. For the NHID and MCSD, the AUROC was 0.680 and 0.665, respectively.

The results of the logistic regression analysis of the NHID and MCSD were as shown in Table 3 and Table 4. In the NHID, The significant predictive factors identified were male, antihypertensive drug, lipid-lowering drug, and walking speed. In this analysis, the odds ratios for male and slow walking speed were 1.824 and 1.367, respectively, indicating positive associations with the diagnosis of T2DM. In contrast, the odds ratios for antihypertensive drug (answer 2: No) and lipid-lowering drug (answer 2: No) were 0.506 and 0.608, respectively, indicating negative association for the diagnosis of T2DM. In the MCSD, the significant predictive factors identified were sex, antihypertensive drug, and eating habits within 2 h before bedtime. The odds ratios for male and eating habits: 2 h before bedtime (answer 2: No) were 1.794 and 3.046, respectively, indicating positive association for the diagnosis of T2DM, whereas the odds ratios for antihypertensive drug (answer 2: No) was 0.601, indicating a negative association for the diagnosis of T2DM. To summarize these results, the common predictive factors were male and antihypertensive drug, while the age-specific predictive factors were lipid-lowering drug, walking speed, and eating habits: 2 h before bedtime.

## 4. Discussion

This study developed predictive models for T2DM using non-invasive parameters obtained from specific health checkups in a regional Japanese population. The models demonstrated moderate predictive ability, with an AUROC of 0.680 for individuals 40–74 years and 0.665 for those aged ≥75 years. The key predictors identified were male sex across both age groups, while slow walking speed and the absence of antihypertensive or lipid-lowering drug use were specific to participants aged 40–74 years. Among those aged ≥75 years, no eating habits within two hours before bedtime and the absence of antihypertensive drug use were significant predictors.

The AUROC in our models was lower than in previous studies. Heianza et al., using a Japanese cohort of 7654 non-diabetic individuals aged 40–75 years, reported that a non-invasive risk score based on age, sex, family history of diabetes, smoking status, and body mass index demonstrated moderate discriminative ability for five-year incident type 2 diabetes (AUROC 0.708) [13]. Nanri et al., developed a large-scale prediction model for T2DM using non-invasive and invasive parameters [8]. Their non-invasive model achieved an AUROC of 0.717 in the derivation cohort and 0.734 in the validation cohort. Additionally, an invasive model including fasting plasma glucose and HbA1c improved the AUROC to 0.893 and 0.882, respectively. In Nanri et al.’s study, their non-invasive risk model included sex, age, body mass index, waist circumference, hypertension, and smoking status. Although the specific set of predictors differed slightly, most of these variables were also incorporated into our models. Importantly, their validation dataset comprised approximately 12,500 participants, which is considerably larger than the sample size in our study. Additionally, Xu et al., using a Japanese population-based cohort of 10,986 individuals, reported that a non-invasive risk model incorporating sex, body mass index, family history of diabetes, and diastolic blood pressure demonstrated moderate discriminative ability for five-year incident type 2 diabetes (AUROC, 0.643) [11]. Similarly, Kawasoe et al., using Japanese health checkup data from 31,084 participants, reported that a non-invasive risk prediction equation incorporating age, sex, body mass index, and hypertension demonstrated modest discriminative ability for five-year incident diabetes (AUROC 0.70) [14]. Previous studies and our research results are summarized in Table 5. In summary, our model was developed using a small sample size, whereas the previous study constructed its model based on a substantially larger cohort with a limited set of predictive variables, and consequently achieved superior predictive performance. In contrast, our study demonstrated that even with a relatively small and region-specific sample, a model based solely on easily obtainable health checkup questionnaire items can achieve moderate predictive performance. This finding emphasizes the potential contribution of our approach to the development of predictive models that reflect regional characteristics.

The logistic regression analyses revealed two predictive factors common to both the NHID and the MCSD: sex and antihypertensive drugs. This finding is consistent with previous studies that identified these variables as risk factors for diabetes [8]. A lipid-lowering drug was identified as a predictor in the NHID but not in the MCSD. Dyslipidemia has been reported to increase the risk of diabetes among individuals aged 40–54, 55–64, and 65–74 years, but it was not a significant factor among those aged 75 years and older [15]. In the MCSD, an absence of eating habits within two hours before bedtime was identified as a predictor. A previous study revealed that eating four meals per day is associated with a lower risk of type 2 diabetes compared with eating three meals per day [16]. This association was significant for individuals with a BMI < 25 kg/m^2^ but not for those with a BMI ≥ 25 kg/m^2^, and age-specific effects were not examined [17]. Although the present study considered the number of meals, it did not account for meal content. Several studies have explored the relationship between diabetes onset and meal composition, nutrient intake, and related factors. Morimoto et al. analyzed diabetes incidence and eating habits in farming villages in Nagano Prefecture, Japan, and found that higher intakes of vegetables, potatoes, seaweed, fruits, and soybean products were associated with a reduced risk of diabetes [16]. Kimura et al. reported that higher dietary fiber intake in the general Japanese population was linked to a lower risk of type 2 diabetes [18]. Collectively, these findings suggest that more detailed analyses of meal content and nutrient composition may reveal different risk factors across narrower age categories.

Our model was developed using data obtained from a specific region, which may limit its generalizability and model performance. In Japan, all municipalities maintain not only information comparable to that used this study, but also resident-level administrative and socioeconomic data (e.g., income, occupation, household composition, and residential environment). Federated learning offers a privacy-preserving framework to train models collaboratively across municipalities while keeping resident-level data local, which may improve predictive performance [19,20]. Moreover, multi-region longitudinal data with outcome statuses may enable more precise prediction. For example, DeepTrace is a graph neural network (GNN)-based framework that leverages a contact network structure and transmission trajectories to identify superspreaders, while updating its estimates as new tracing information accumulates [21]. Similarly, modeling resident-level data, including annual health checkups, daily life indicators, and healthcare utilization, as longitudinal trajectories and updating risk estimates as observations accrue may further extend our approach toward more powerful predictive models. Realizing such multi-site and individual-level, trajectory-based modeling, however, requires addressing several practical challenges. Specifically, learning from multi-site, high-dimensional data poses well-recognized challenges, including structural heterogeneity and increased complexity in data processing and modeling [22]. Addressing these issues typically requires robust computing environments and well-designed data platforms.

As several limitations of the present study, the data obtained from the questionnaire forms from specific health checkups are self-reported. For example, certain question items had no clear standards for frequency and quantity; these items include “My walking speed is faster compared with persons of the same sex who are roughly my age”; “Compared with others, I eat at a faster speed”; and “When sleeping, I get adequate rest.” This lack of clarity in standards for responses could introduce subjectivity and potential bias in the reported data. This study was based on data from a single municipality in Hokkaido, Japan, and the findings may not be generalizable to other regions. Regional variation in seasonal conditions, dietary patterns, and exercise habits may result in different contextual meanings for identical questionnaire responses. For instance, the types of foods typically consumed within two hours before bedtime may vary across regions. Finally, this study attempted to develop a predictive model using a relatively small sample size and achieved moderate predictive performance. Incorporating resident information held by each municipality into collaborative healthcare learning frameworks, while preserving privacy, has the potential to enhance model performance and support the development of more robust predictive models.

## 5. Conclusions

This study developed predictive models for T2DM using community-based health checkup and claims data. Models based on non-invasive parameters achieved moderate performance (AUROC = 0.660 and 0.618). Key predictive factors included sex, antihypertensive drugs, lipid-lowering drugs, walking speed, and eating habits before bedtime. These findings suggest the potential for more advanced utilization of routinely collected health checkup data in regional settings.

## Figures and Tables

**Table 1:** Health checkup questionnaire.

Items	Answer
Current use of antihypertensive medication	① Yes ② No
Current use of cholesterol-lowering medication	① Yes ② No
Currently, have the habit of smoking. (Note: “Those habitually smoking at present” refers to individuals who have smoked a total of 100 cigarettes or more, or have smoked for 6 months or longer, including those who have smoked in the last month.)	① Yes ② No
Has gained more than 10 kg since the age of 20	① Yes ② No
Engages in moderate exercise, causing sweating for at least 30 min, two or more times a week, continuously for one year or more.	① Yes ② No
Engages in walking or equivalent physical activity for at least one hour per day in daily life.	① Yes ② No
Walks at a faster pace compared to individuals of the same gender and roughly the same age.	① Yes ② No
Experienced a weight change of ±3 kg or more in the past year.	① Yes ② No
Eats at a faster pace compared to others.	① Fast ② Average ③ Slow
Eats dinner within 2 h before bedtime at least three times a week.	① Yes ② No
Has snacks (supper other than the three main meals) at least three times a week after dinner.	① Yes ② No
Skips breakfast at least three times a week.	① Yes ② No
Frequency of consuming alcoholic beverages (sake, shochu, beer, Western liquor, etc.)	① Every day ② Occasionally ③ Almost never (Cannot drink)
Daily alcohol consumption on drinking days Approximate equivalents: Guidelines for 1 cup (180 mL) of sake: 1 medium bottle of beer (about 500 mL) 80 mL of 35% alcohol shochu 1 double shot of whiskey (60 mL) 2 glasses of wine (240 mL)	① Less than 1 cup (180 mL) ② 1 to less than 2 cup ③ 2 to less than 3 cup ④ 3 cup or more
Getting sufficient rest through sleep.	① Yes ② No
Are you considering making improvements to lifestyle habits such as exercise and dietary choices?	① No intention to improve ② Intend to improve (generally within 6 months) ③ Intend to improve soon (generally within 1 month) and have started gradually ④ Already working on improvement (less than 6 months) ⑤ Already working on improvement (6 months or more)
If there is an opportunity to receive health guidance on improving lifestyle habits, would you take advantage of it?	① Yes ② No

**Table 2:** Characteristics of the participants.

	National Health Insurance Dataset			Medical Care System Dataset		
	With T2DM (n = 285)	Without T2DM (n = 665)	*p*-Value	With T2DM (n = 124)	Without T2DM (n = 289)	*p*-Value
BMI, mean (SD)	24.72 (3.95)	23.35 (3.42)	0.007	23.05 (3.53)	22.88 (3.05)	0.637
Waist, mean (SD)	85.57 (10.12)	83.38 (9.01)	0.002	83.00 (9.73)	82.41 (8.30)	0.561
Male, n (%)	177 (62.1)	306 (46.0)	<0.001	80 (64.5)	149 (51.6)	0.015
Antihypertensive drug, yes, n (%)	165 (57.9)	253 (38.0)	<0.001	78 (62.9)	144 (49.8)	0.015
Lipid-lowering drug, yes, n (%)	127 (44.5)	214 (32.2)	<0.001	54 (43.5)	99 (34.3)	0.073
Smoking, yes, n (%)	55 (19.3)	106 (15.9)	0.206	9 (7.3)	13 (4.5)	0.252
Weight change from age 20, y, n (%)	127 (44.6)	260 (39.1)	0.116	34 (27.4)	69 (23.9)	0.446
Exercise habit of 30 min or more, yes, n (%)	125 (43.9)	255 (38.3)	0.112	56 (45.2)	131 (45.3)	0.975
Walking or physical activity, yes, n (%)	133 (46.7)	308 (46.3)	0.921	68 (30.6)	154 (53.3)	0.772
Walking speed, yes, n (%)	137 (48.1)	371 (55.8)	0.288	55 (44.4)	129 (44.6)	0.958
Weight change over 1 year, yes, n (%)	76 (26.7)	142 (21.4)	0.074	24 (19.4)	39 (13.5)	0.129
Speed of eating, yes, n (%)			0.272			0.507
Fast	96 (33.7)	207 (31.1)		23 (18.5)	45 (15.6)	
Normal	168 (58.9)	423 (63.6)		84 (67.7)	212 (73.4)	
Slow	21 (7.4)	35 (5.3)		17 (13.7)	32 (11.1)	
Eating habits 2 h before bedtime, yes, n (%)	40 (14.0)	89 (13.4)	0.788	14 (11.3)	44 (15.2)	0.292
Snack after dinner, yes, n (%)	44 (15.4)	95 (14.3)	0.645	5 (4.0)	27 (9.3)	0.064
Eating habits of skipping breakfast, yes, n (%)	27 (9.5)	49 (7.4)	0.273	6 (4.8)	8 (2.8)	0.287
Drinking habits, yes, n (%)			0.234			0.518
Everyday	93 (32.6)	181 (27.2)		26 (21.0)	48 (16.6)	
Sometimes	96 (33.7)	247 (37.1)		35 (28.2)	80 (27.7)	
Almost no	96 (33.7)	237 (35.6)		63 (50.8)	161 (55.7)	
Alcohol consumption			0.681			0.793
Less than one drink	179 (62.8)	431 (64.8)		99 (79.8)	240 (83.0)	
Between 1 and 2 drinks	65 (22.8)	131 (19.7)		18 (145)	38 (13.1)	
Between 2 and 3 drinks	35 (12.3)	84 (12.6)		6 (4.8)	10 (3.5)	
More than 3 drinks	6 (2.1)	19 (2.8)		1 (0.8)	1 (0.3)	
Sleep (adequate rest), yes, n (%)	219 (76.8)	519 (78.0)	0.683	97 (78.2)	242 (83.7)	0.181
Intention to improve lifestyle habits, yes, n (%)			0.266			0.961
No plan on improving	67 (23.5)	179 (26.9)		35 (28.2)	81 (28.0)	
Plan on improving within 6 months	62 (21.8)	167 (25.1)		12 (9.7)	33 (11.4)	
Plan on within 1 month and start little by little	30 (10.5)	76 (11.4)		18 (14.5)	39 (13.5)	
In the process of improving for 6 months	35 (12.3)	73 (11.0)		13 (10.5)	35 (12.1)	
In the process of improving for over 6 months	91 (31.9)	170 (25.6)		46 (37.1)	101 (34.9)	
Request for health guidance, yes, n (%)	139 (48.8)	311 (46.8)	0.571	55 (44.4)	138 (47.8)	0.526

T2DM, type 2 diabetes mellitus.

**Table 3:** Logistic regression analysis results in National Health Insurance Dataset.

	Coefficient	SE	OR	95% CI
Intercept	−1.301	1.163		
BMI	−0.028	0.043	0.972	0.893–1.059
Waist	0.023	0.016	1.023	0.990–1.056
Male	0.601	0.172	1.824	1.301–2.556
Antihypertensive drug, no	−0.681	0.157	0.506	0.372–0.688
Lipid-lowering drug, no	−0.498	0.161	0.608	0.444–0.833
Smoking, no	−0.058	0.205	0.944	0.632–1.409
Weight change from age 20, no	0.181	0.192	1.198	0.822–1.747
Exercise habit of 30 min or more, no	−0.179	0.189	0.836	0.577–1.211
Walking or physical activity, no	−0.053	0.181	0.949	0.665–1.353
Walking speed, no	0.313	0.157	1.367	1.005–1.859
Weight change over 1 year, no	−0.163	0.186	0.849	0.590–1.223
Speed of eating, Slow	ref			
Fast	−0.260	0.329	0.771	0.405–1.470
Normal	−0.347	0.314	0.707	0.382–1.309
Snack after dinner, no	0.037	0.228	1.037	0.663–1.622
Eating habits 2 h before bedtime, no	−0.083	0.221	0.921	0.596–1.421
Eating habits, no	−0.524	0.282	0.592	0.341–1.029
Drinking habits, Almost no	ref			
Everyday	0.150	0.231	1.162	0.739–1.827
Sometimes	−0.100	0.198	0.905	0.613–1.334
Alcohol consumption, More than 3 drinks	ref			
Less than one drink	0.744	0.524	2.104	0.753–5.880
Between 1 and 2 drinks	0.756	0.528	2.129	0.756–5.994
Between 2 and 3 drinks	0.336	0.543	1.399	0.483–4.051
Sleep (adequate rest), no	0.126	0.185	1.135	0.789–1.632
Intention to improve lifestyle habits, In the process of improving for over 6 months.	ref			
No plan on improving	−0.266	0.215	0.767	0.503–1.168
Plan on improving within 6 months	−0.378	0.225	0.685	0.441–1.066
Plan on improving within 1 month and start little by little	−0.419	0.280	0.658	0.380–1.138
In the process of improving for 6 months	−0.174	0.259	0.841	0.506–1.397
Request for health guidance, no	−0.142	0.156	0.867	0.638–1.178

SE, standard error; OR, odds ratio; CI, confidence interval, BMI, body mass index.

**Table 4:** Logistic regression analysis results in Medical Care System dataset.

	Coefficient	SE	OR	95% CI
Intercept	1.335	2.343		
BMI	−0.016	0.071	0.985	0.857–1.131
Waist	−0.003	0.026	0.997	0.948–1.049
Male	0.585	0.268	1.794	1.061–3.035
Antihypertensive drug, no	−0.509	0.243	0.601	0.373–0.968
Lipid-lowering drug, no	−0.432	0.248	0.649	0.400–1.055
Smoking, no	−0.450	0.496	0.638	0.241–1.685
Weight change from age 20, no	−0.129	0.314	0.879	0.474–1.627
Exercise habit of 30 min or more, no	0.088	0.284	1.092	0.626–1.905
Walking or physical activity, no	−0.263	0.273	0.769	0.450–1.313
Walking speed, no	−0.074	0.244	0.929	0.576–1.498
Weight change over 1 year, no	−0.508	0.330	0.602	0.315–1.148
Speed of eating, Slow	ref			
Fast	−0.038	0.435	0.963	0.411–2.258
Normal	−0.408	0.358	0.665	0.330–1.342
Snack after dinner, no	0.324	0.356	1.383	0.689–2.778
Eating habits 2 h before bedtime, no	1.114	0.561	3.046	1.015–9.146
Eating habits, no	−0.470	0.637	0.625	0.179–2.177
Drinking habits, Almost no	ref			
Everyday	0.061	0.362	1.063	0.523–2.163
Sometimes	−0.085	0.312	0.918	0.498–1.692
Alcohol consumption, More than 3 drinks	ref			
Less than one drink	−0.991	1.534	0.371	0.018–7.506
Between 1 and 2 drinks	−1.080	1.558	0.339	0.016–7.191
Between 2 and 3 drinks	−1.030	1.624	0.357	0.015–8.610
Sleep (adequate rest), no	0.421	0.304	1.523	0.840–2.762
Intention to improve lifestyle habits, In the process of improving for over 6 months.	ref			
No plan on improving	−0.039	0.295	0.962	0.540–1.715
Plan on improving within 6 months	−0.522	0.443	0.593	0.249–1.413
Plan on improving within 1 month and start little by little	−0.070	0.387	0.933	0.437–1.992
In the process of improving for 6 months	−0.197	0.399	0.821	0.376–1.795
Request for health guidance, no	0.142	0.240	1.153	0.720–1.847

SE, standard error; OR, odds ratio; CI, confidence interval, BMI, body mass index.

**Table 5:** Comparison of non-invasive T2DM prediction models.

Study	Year	Derivation Cohort	Age	Prediction Horizon	AUROC	Validation	Outcome Definition
Heianza, Y. et al. [13]	2012	7654	40–75	5-year	0.708	External	FPG, HbA1c, self-reported (diagnosed)
Nanri, A. et al. [8]	2015	24,950	≥30	3-year	0.734	Internal	HbA1c, FPG, random plasma glucose, medical treatment
Xu, J. et al. [11]	2024	10,986	46–75	5-year	0.643	External	HbA1c, FPG, random plasma glucose level, self-reported (diagnosed or treatment)
Kawasoe, S. et al. [14]	2025	15,542	30–69	5-year	0.710	Internal	Use antidiabetic agents or HbA1c
Ours (40–74)	-	1363	40–74	2-year	0.680	None	Claims data
Ours (≥75)	-	950	≥75	2-year	0.665	None	Claims data

AUROC, area under the receiver operating characteristic curve.

## Data Availability

The data used in this study are not permitted to be shared with third parties.

## Supplementary Materials

The following supporting information can be downloaded at https://www.mdpi.com/article/10.3390/bioengineering13020194/s1, Figure S1: Participant extraction flow in the National Health Insurance dataset; Figure S2: Participant extraction flow in the Medical Care System dataset.

## Abbreviations

The following abbreviations are used in this manuscript:

AUROC	Area Under the Receiver Operating Characteristic Curve
BMI	Body Mass Index
FY	Fiscal Year
HbA1c	Hemoglobin A1c
MCSD	Medical Care System Dataset (for the Elderly Aged 75 and Over)
NCD	Non-Communicable Disease
NHID	National Health Insurance Dataset
T2DM	Type 2 Diabetes Mellitus
