# Supplementary material for: Development of a Type 2 Diabetes Prediction Model Using Specific Health Checkup Data and Extraction of Predictive Factors
Source: Bioengineering (Basel). 2026 Feb 9;13(2):194. doi: 10.3390/bioengineering13020194 (PMC12938782; doi:10.3390/bioengineering13020194)

Figure S1 Participants extraction flow in National Health Insurance dataset

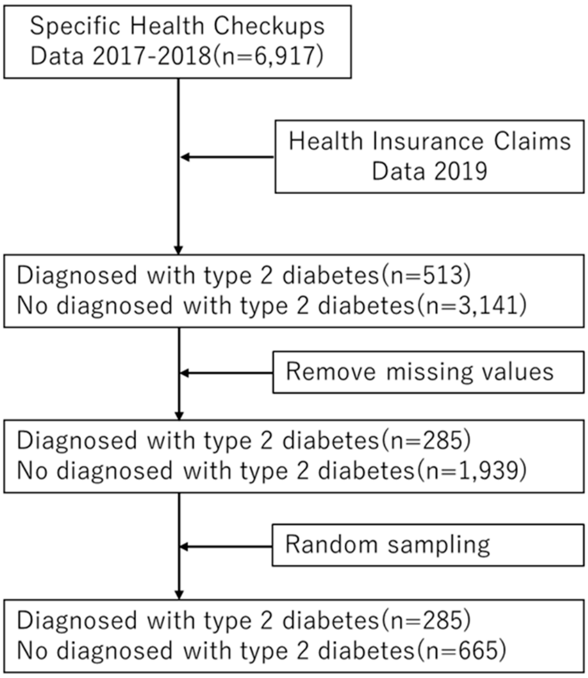

Fig. S2　Participants extraction flow in Medical Care System dataset

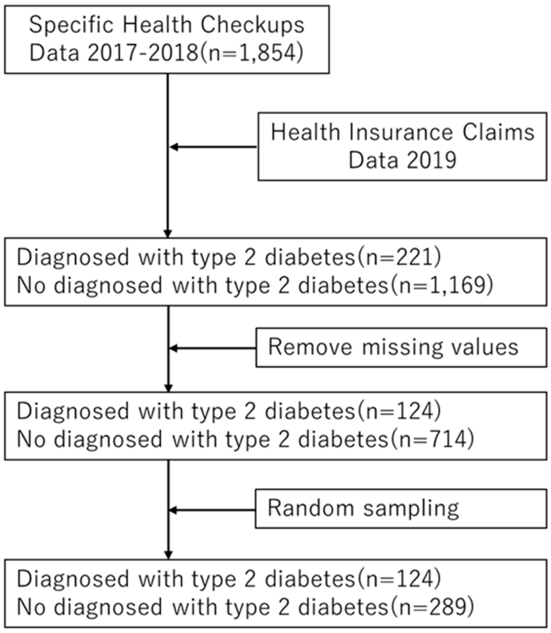

Supplement: Supplementary file 1 [file bioengineering-13-00194-s001.zip › bioengineering-4015378-supplementary.pdf]
